# Disease burden in patients with acute hepatic porphyria: experience from the phase 3 ENVISION study

**DOI:** 10.1186/s13023-022-02463-x

**Published:** 2022-08-26

**Authors:** Bruce Wang, Paolo Ventura, Kei-ichiro Takase, Manish Thapar, David Cassiman, Ilja Kubisch, Shangbin Liu, Marianne T. Sweetser, Manisha Balwani

**Affiliations:** 1 Division of Gastroenterology, Department of Medicine, University of California San Francisco, San Francisco, CA 94143 USA; 2 University of Modena and Reggio Emilia, Modena, Italy; 3 Iizuka Hospital, Iizuka, Japan; 4 Thomas Jefferson University, Philadelphia, PA USA; 5 University Hospital Leuven, Leuven, Belgium; 6 Klinikum Chemnitz, Chemnitz, Germany; 7 Alnylam Pharmaceuticals, Cambridge, MA USA; 8 Icahn School of Medicine at Mount Sinai, New York, NY USA

**Keywords:** Acute hepatic porphyria, Givosiran, Disease burden, Porphyria attack, Chronic symptoms, Quality of life

## Abstract

**Background:**

Acute hepatic porphyria (AHP) is a family of four rare genetic diseases, each involving deficiency in a hepatic heme biosynthetic enzyme. Resultant overproduction of the neurotoxic intermediates δ-aminolevulinic acid (ALA) and porphobilinogen (PBG) leads to disabling acute neurovisceral attacks and progressive neuropathy. We evaluated the AHP disease burden in patients aged ≥ 12 years in a post hoc analysis of the Phase 3, randomized, double-blind, placebo-controlled ENVISION trial of givosiran (NCT03338816), an RNA interference (RNAi) therapeutic that targets the enzyme ALAS1 to decrease ALA and PBG production. We analyzed baseline AHP severity via chronic symptoms between attacks, comorbidities, concomitant medications, hemin-associated complications, and quality of life (QOL) and evaluated givosiran (2.5 mg/kg monthly) in patients with and without prior hemin prophylaxis on number and severity of attacks and pain scores during and between attacks.

**Results:**

Participants (placebo, n = 46; givosiran, n = 48) included patients with low and high annualized attack rates (AARs; range 0–46). At baseline, patients reported chronic symptoms (52%), including nausea, fatigue, and pain; comorbidities, including neuropathy (38%) and psychiatric disorders (47%); concomitant medications, including chronic opioids (29%); hemin-associated complications (eg, iron overload); and poor QOL (low SF-12 and EuroQol visual analog scale scores). A linear relationship between time since diagnosis and AAR with placebo suggested worsening of disease over time without effective treatment. Givosiran reduced the number and severity of attacks, days with worst pain scores above baseline, and opioid use versus placebo.

**Conclusions:**

Patients with AHP, regardless of annualized attack rates, have considerable disease burden that may partly be alleviated with givosiran.

## Background

Acute hepatic porphyria (AHP) is a family of rare genetic diseases, each arising from a deficiency in an enzyme involved in hepatic heme biosynthesis [1]. These enzyme defects cause depletion of free heme and increase the demand for hepatic heme, resulting in up-regulation of the ALAS1 enzyme and overproduction of the toxic heme intermediates delta-aminolevulinic acid (ALA) and porphobilinogen (PBG) [2]. Accumulation of ALA and PBG is thought to cause injury primarily to the nervous system, as well as to other organs, such as the liver and kidneys [2–4]. Of the four subtypes of AHP, the most common is acute intermittent porphyria (AIP) [1]. AHP manifests predominantly in females between the ages of 20 and 40 years [5, 6], and the overall symptomatic prevalence is 1 per 100,000 persons [7, 8]. While most symptomatic patients experience only a few attacks in their lifetime, up to 8% have recurrent attacks (≥ 4 attacks per year) [9].

AHP is a multisystem disease characterized by disabling acute neurovisceral attacks, most often including severe abdominal pain, vomiting, muscle weakness, hypertension, and changes in mental status [2]. Chronic manifestations occur between attacks and progress over the disease course [2, 6, 10, 11]. In a multinational natural history study of 112 patients with AHP experiencing recurrent attacks (EXPLORE), 77% of attacks required treatment at a healthcare facility and/or administration of hemin [11]. Attacks not treated promptly or that frequently recur may lead to progressive or irreversible neuropathy and prolonged debilitation [2, 11]. Approximately 59% of patients with recurrent attacks will develop chronic kidney disease [12]. Recurrent AHP also has a long-term impact on mental functioning, affecting activities of daily living [6, 10, 13]. Patients report diminished quality of life (QOL) and significant economic burden [11, 14].

Early treatment is important to prevent disease progression [5, 15]. There is a need for treatments that not only reduce or eliminate acute attacks, but also improve chronic manifestations of recurrent AHP. Prevention strategies include identifying and avoiding triggers that may precipitate acute attacks [16, 17]. However, some triggers, such as stress, may not always be avoidable [17, 18]. Furthermore, many patients continue to experience chronic symptoms between attacks [6, 10, 11]. Opioids are used to manage abdominal, limb, and back pain [17], but patients receiving opioids may develop somnolence, addiction, tolerance, and hyperalgesia [19].

Intravenous (IV) hemin is the standard of care for confirmed acute attacks [2, 20, 21]. Hemin is thought to work by decreasing levels of ALAS1, thereby reducing the production of toxic heme precursors [22–24]. Although hemin is not indicated for prophylaxis [22, 23], it is often used off-label for this purpose [11]. Some patients have reported recurrent attacks despite weekly prophylactic hemin administration [25]. Side effects and complications associated with hemin use include headache, infusion-site reactions, and phlebitis, and chronic complications, such as tachyphylaxis, coagulation abnormalities, venous damage, and secondary iron overload [25, 26]. Chronic hemin use is also associated with liver inflammation and fibrosis [25]. Patients may also have complications from the indwelling central venous catheter required to administer hemin on a regular basis, including catheter occlusion, bacteremia, and thromboembolism [25].

Liver transplantation is considered a last resort for patients with severe recurrent attacks that do not respond to hemin [5, 26, 27]. It is not suitable for most patients, and there is a shortage of donors [11, 28]. It also has a significant risk of morbidity and mortality that is comparable to that faced by patients who received transplants for other metabolic diseases [11, 25, 27].

Thus, treatment options for recurrent AHP were limited before the approval of givosiran, a subcutaneously administered RNA interference (RNAi) therapeutic that specifically targets ALAS1 messenger RNA in the liver to reduce production of ALA and PBG [29, 30]. Givosiran is indicated for the treatment of AHP in adults (United States, Brazil, Canada) and adolescents aged 12 years and older (European Economic Area, United Kingdom, Switzerland, Japan) [31–34] based on the results of the phase 3 ENVISION trial [35]. In ENVISION (NCT03338816), givosiran treatment was associated with a 74% reduction in the mean annualized rate of composite porphyria attacks (AAR) in patients with AIP compared with placebo (3.2 vs. 12.5, respectively; *P* < 0.001) [35]. Givosiran treatment also reduced urinary ALA and PBG levels and hemin use and was associated with improvement in pain and QOL assessment scores. Continued treatment through month 24 led to sustained reductions in ALA and PBG levels, porphyria attacks, and hemin use, and to further improvements in QOL and other patient-reported outcomes [36]. The proportion of patients with no attacks increased over time, with 83% of patients who received continuous givosiran treatment remaining attack-free during the 3 months prior to Month 24 [36]. The safety profile of givosiran was acceptable, and most adverse events (AEs) were mild or moderate in severity [35, 36].

This post hoc analysis used data from the ENVISION trial to evaluate the disease burden associated with AHP, including patients with a low rate of acute attacks (< 7 attacks or < 12 attacks in the previous 12 months among patients who were and were not receiving hemin at baseline, respectively). The efficacy of givosiran in patients with and without prior hemin use was also assessed, including patient-reported pain scores during and between attacks.

## Methods

### ENVISION trial design

The study population was defined as patients aged ≥ 12 years with a diagnosis of AHP, an elevated level of urinary ALA or PBG (≥ 4 times the upper limit of normal), and either a confirmed pathogenic mutation associated with AHP or biochemical and clinical criteria consistent with a diagnosis of AHP [35]. Patients were also required to have experienced ≥ 2 porphyria attacks requiring hospitalization, urgent health care, or IV administration of hemin at home in the previous 6 months [35]. Details regarding study subjects and methodology of the ENVISION study have been previously reported [35].

Thirty-six study sites in 18 countries participated in the study [35]. Patients were randomized to receive monthly subcutaneous givosiran 2.5 mg/kg or matching placebo for 6 months [35]. Randomization was stratified according to AHP subtype, previous use or nonuse of hemin prophylaxis, and a low or high AAR in the previous 12 months (< 7 attacks [low] vs. ≥ 7 attacks [high] among patients who were receiving hemin prophylaxis at baseline and < 12 attacks [low] vs. ≥ 12 attacks [high] among those who were not receiving hemin prophylaxis) [35].

### Outcome measures

The primary endpoint was AAR (requiring hospitalization, urgent health care, or IV administration of hemin at home) among patients with AIP [35]. Secondary endpoints included AAR among all patients with AHP; rate of administered hemin doses; daily worst scores for pain, fatigue, and nausea; and QOL assessments in patients with AIP. Exploratory endpoints included analgesic usage (opioid and non-opioid).

Daily worst pain and daily worst fatigue scores were measured using the Brief Pain Inventory-Short Form (BPI-SF) and Brief Fatigue Inventory-Short Form (BFI-SF) numerical rating scales (NRS), respectively. An NRS was also used for daily worst nausea score. All NRS ranged from 0 to 10, with higher scores indicating more severe symptoms; scores were captured using a daily eDiary from screening through Month 12.

QOL assessments included the 12-Item Short-Form Health Survey, version 2 (SF-12), and the EuroQol-5 Dimension-5 Level Questionnaire (EQ-5D-5L) and were collected throughout the study. The SF-12 generates 8 domains of functional health and well-being, including bodily pain, in addition to physical component summary (PCS) and mental component summary (MCS) scores. Low scores on the PCS indicate limitations in physical functioning, high bodily pain, and poor general health. The EQ-5D-5L assesses 5 dimensions, as well as patient’s global impression of their overall health on a visual analog scale (EQ VAS), which ranges from 0 (worst possible health) to 100 (best possible health).

### Post hoc analyses

A review of ENVISION trial data was undertaken to fully examine the severity of AHP disease burden at baseline, including the prevalence of chronic symptoms, comorbidities, concomitant medications, prophylactic hemin use and associated complications, and patient QOL. Additional measurements included lost days of work due to AHP and hours of caregiver support required. The association between time since diagnosis and AAR was also examined. Furthermore, post hoc analyses were undertaken to examine the effects of givosiran treatment on the number and severity of attacks in patients with AHP according to prior hemin prophylaxis, and on daily worst pain scores and analgesic use during attack-free periods. These analyses were not protocol-defined, and therefore no formal statistical comparisons were undertaken.

## Results

A total of 94 patients with AHP were enrolled in the ENVISION trial, of whom 46 were assigned to receive placebo and 48 to receive givosiran [35]. The median (range) age was 37.5 (19‒65) years, and the mean (SD) years since diagnosis was 9.7 (10.0) [35]. The median (range) AAR in the 6 months prior to study randomization was 8.0 (0–46) [35].

At baseline, 52% of patients were experiencing chronic symptoms of porphyria, defined as symptoms between attacks daily or on most days [35]. Chronic symptoms included nausea, fatigue, and pain (Table 1). Opioid analgesics were used daily or on most days between attacks in 29% of patients [35]. Mean daily worst fatigue score was above 4, on a numerical rating scale of 0–10, in both treatment groups.

**Table 1:** Chronic symptoms in ENVISION trial participants at baseline

Characteristic	Placebo (n = 46)	Givosiran (n = 48)	Total (N = 94)
Prior chronic symptoms, n (%)	26 (57)	23 (48)	49 (52)
Prior chronic opioid use, n (%)	13 (28)	14 (29)	27 (29)
Nausea symptoms (medical history), n (%)	10 (22)	7 (15)	17 (18)
Fatigue (medical history), n (%)	4 (9)	1 (2)	5 (5)
Daily worst nausea score, weekly mean (SD)	1.9 (1.8)	1.6 (1.7)	1.7 (1.8)
Daily worst fatigue score, weekly mean (SD)	4.7 (2.3)	4.1 (2.6)	4.4 (2.5)
Daily worst pain score, weekly mean (SD)	3.7 (2.2)	3.0 (2.3)	3.3 (2.3)
Bodily pain domain score (SF-12), mean (SD)	34.4 (9.0)	37.6 (9.9)	36.0 (9.6)

*SF-12* 12-Item Short-Form Health Survey, version 2

Patients had a poor QOL at baseline, as measured using the SF-12 PCS, SF-12 MCS, and EQ VAS (Table 2). While the study population was of working age (19–65 years), only 44% of patients were employed. Those who were employed missed a mean of 6 days and 3 days within the 4 weeks prior to their baseline visit in the placebo and givosiran group, respectively.

**Table 2:** Quality of life in ENVISION trial participants at baseline

Assessment, Mean (SD)	Placebo (n = 46)	Givosiran (n = 48)
SF-12 PCS	38.1 (9.8)	39.5 (9.8)
SF-12 MCS	41.8 (10.3)	39.9 (8.3)
EQ VAS	64.3 (19.6)	62.6 (22.6)
Employed in past 4 weeks, n	21	20
Days of work missed in past 4 weeks	6.1 (6.5)	3.3 (3.5)
Hours of caregiver support in past week	11.3 (28.1)	12.5 (32.3)

*EQ VAS* EuroQol visual analog scale, *MCS* mental component summary, *PCS* physical component summary, *SF-12* 12-Item Short-Form Health Survey, version 2

**Fig. 1:**
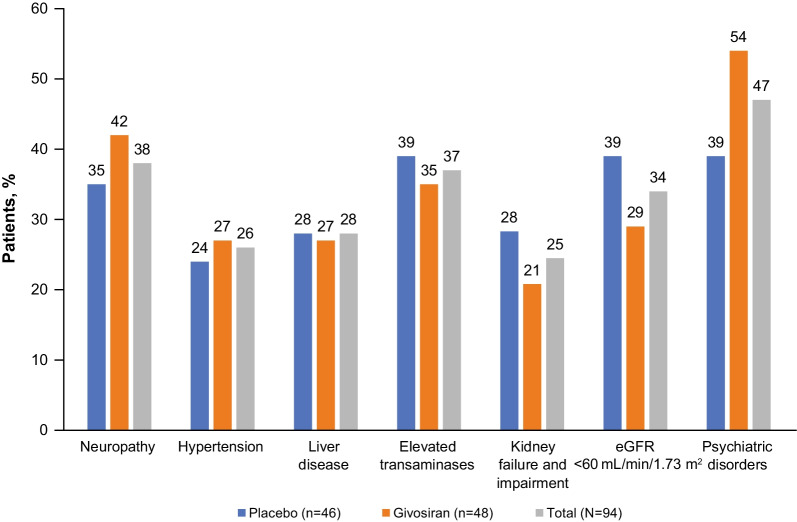
Comorbidities in ENVISION trial participants at baseline

A high proportion of patients had comorbidities, including peripheral neuropathy, hypertension, liver and kidney disease, and psychiatric disorders (Fig. 1). Sensory neuropathy was present in 19% of patients, motor neuropathy in 22% of patients, and autonomic neuropathy in 3% of patients. Overall rates of depression, anxiety, and insomnia were 27%, 23%, and 18%, respectively. A total of 29% of patients reporting being moderately, severely, or extremely anxious or depressed. Mean score for the SF-12 MCS was lower (40.9) than the population norm (50.0) [37], suggesting that AHP has a negative impact on mental health. Many patients were also taking concomitant medications that coincide with common comorbidities, including antidepressants, antihypertensives, antiemetics, and opioids (Table 3).

**Table 3:** Concomitant antidepressants, antihypertensives, antiemetics, and analgesics at baseline

	Placebo (n = 46)	Givosiran (n = 48)	Total (N = 94)
Antidepressants			
Benzodiazepine derivatives	10 (22)	10 (21)	20 (21)
Benzodiazepine-related drugs	3 (7)	4 (8)	7 (7)
Other anti-depressants	4 (9)	9 (19)	13 (14)
SSRIs	2 (4)	4 (8)	6 (6)
Antihypertensives			
ACE inhibitors	2 (4)	1 (2)	3 (3)
Angiotensin II receptor antagonists	3 (7)	2 (4)	5 (5)
Beta blocking agents, non-selective	1 (2)	3 (6)	4 (4)
Beta blocking agents, selective	5 (11)	7 (15)	12 (13)
Antiemetics			
5HT3 antagonists	12 (26)	12 (25)	24 (26)
Pain medications			
Natural opium alkaloids	27 (59)	23 (48)	50 (53)
Opioid anesthetics (fentanyl)	2 (4)	0	2 (2)
Opioid/non-opioid combinations	2 (4)	5 (10)	7 (7)
Opium alkaloid derivatives	2 (4)	0	2 (2)
Other opioids	8 (17)	6 (13)	14 (15)
Other analgesics and antipyretics	10 (22)	13 (27)	23 (25)

*SSRI* selective serotonin reuptake inhibitor, *ACE* angiotensin-converting enzyme, *5HT3* 5-hydroxytryptamine

**Fig. 2:**
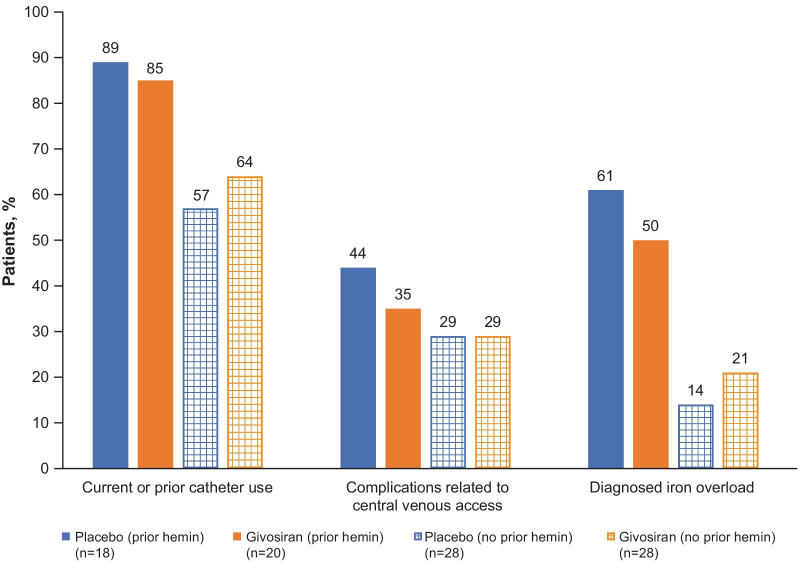
Complications and comorbidities in ENVISION trial participants according to hemin prophylaxis at baseline

A high proportion of patients had received prior hemin prophylaxis (40%) [11], with many experiencing hemin-related complications, as well as ongoing damage caused by AHP (Fig. 2). Complications related to central venous access included thrombosis (8%), infection (18%), and catheter occlusion/malfunction (24%). Disease burden was also high in patients who had not received prior hemin prophylaxis (60%) (Table 4).

**Table 4:** Disease severity in ENVISION trial participants according to hemin prophylaxis at baseline

	No prior hemin prophylaxis		Prior hemin prophylaxis	
	Placebo (n = 28)	Givosiran (n = 28)	Placebo (n = 18)	Givosiran (n = 20)
Historical AAR, median (range)	6.0 (0–46)	8.0 (4–34)	9.0 (4–38)	9.0 (4–32)
Chronic symptoms, n (%)	17 (61)	16 (57)	9 (50)	7 (35)
Chronic opioid use, n (%)	7 (25)	6 (21)	6 (33)	8 (40)
EQ VAS, mean (SD)	63.9 (20.7)	58.4 (23.0)	64.8 (18.4)	68.4 (21.3)
SF-12 PCS, mean (SD)	36.5 (10.5)^a^	39.3 (11.2)	40.5 (8.4)	39.7 (7.8)

*AAR* annualized attack rate, *EQ VAS* EuroQol visual analog scale, *PCS* physical component summary, *SF-12* 12-Item Short-Form Health Survey, version 2

^a^Data missing for 1 patient

**Fig. 3:**
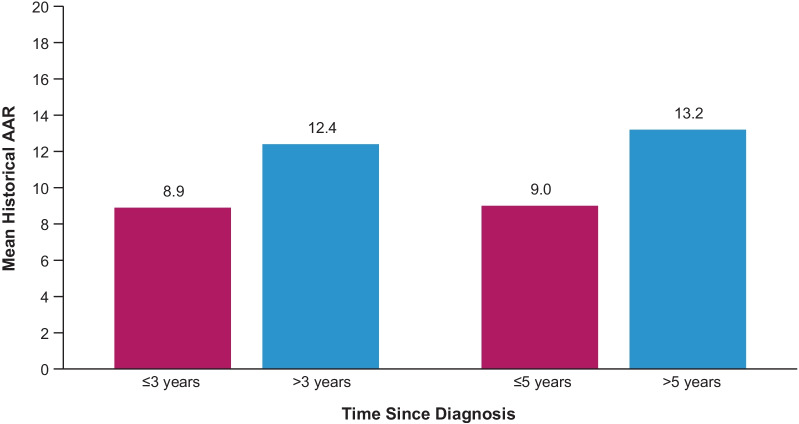
Association between years since diagnosis of AHP and mean historical annualized attack rate during ENVISION trial

There was a linear relationship between longer time since diagnosis of AHP and higher AAR in the placebo group during the 6-month double-blind treatment period of the ENVISION trial (Fig. 3; r = 0.403; *P* < 0.01). This suggests that AHP disease worsening likely occurs over time.

**Fig. 4:**
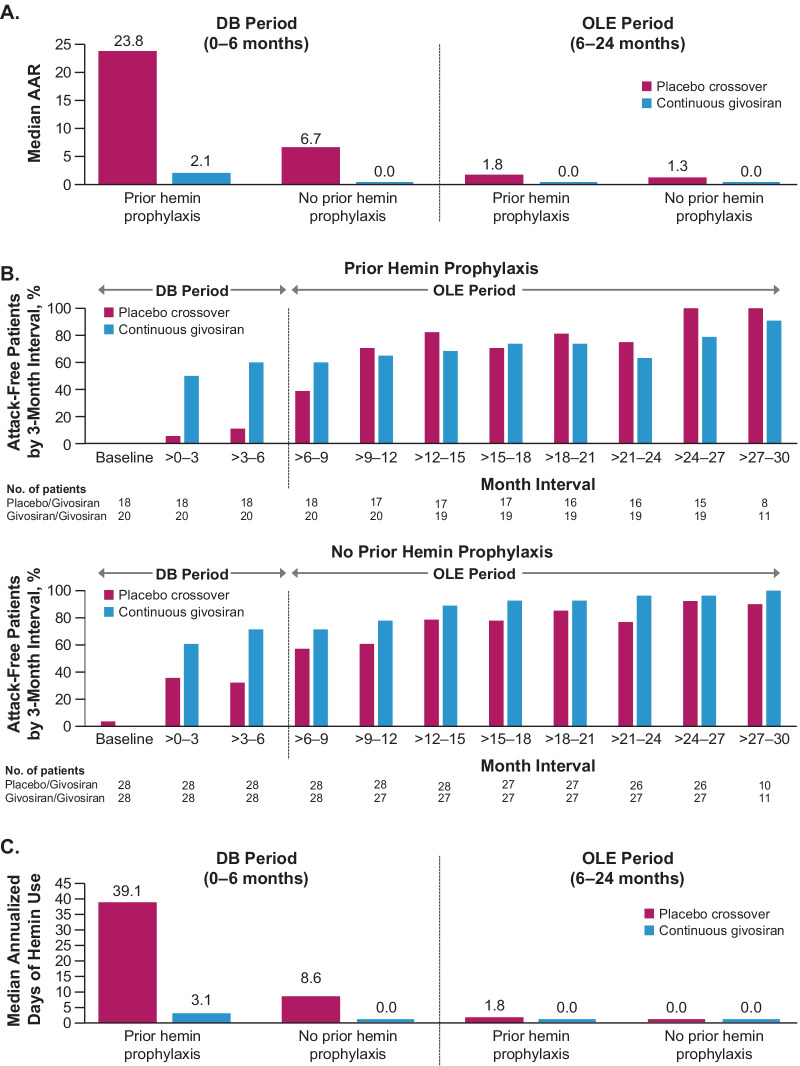
Attack frequency (**A**), proportion of attack-free patients by 3-month interval (**B**), and hemin use (**C**) with long-term givosiran treatment in patients with or without prior hemin prophylaxis use^a^. ^a^Hemin prophylaxis was not allowed during the study; days of hemin use therefore refers only to hemin used to treat attacks

**Fig. 5:**
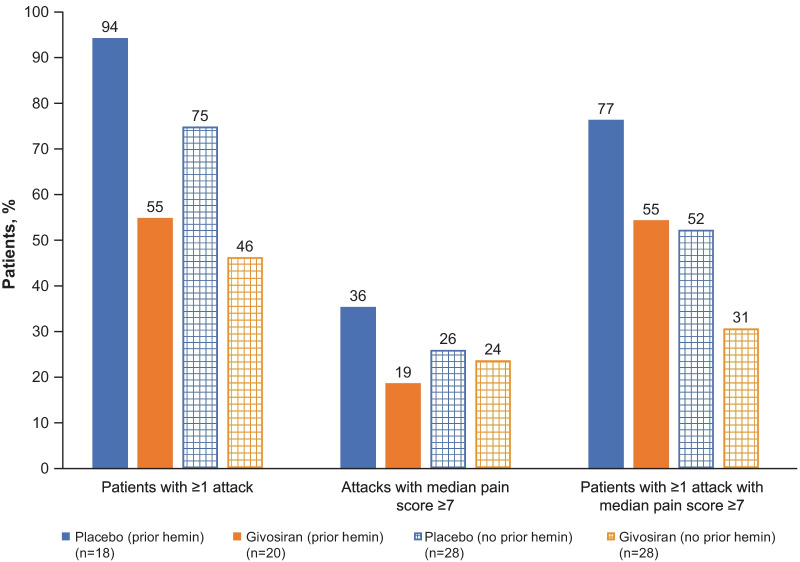
Proportions of patients with ≥ 1 attack at Month 6 in ENVISION trial according to hemin prophylaxis at baseline

The number of attacks, and attack severity, were both reduced in patients with AHP receiving givosiran compared with those receiving placebo, regardless of prior hemin use (Fig. 4). The total number of attacks among those with no prior hemin prophylaxis was 42 in the givosiran group versus 111 in the placebo group. Corresponding numbers among those who did have prior hemin prophylaxis were 48 and 186, respectively. The proportion of patients with ≥ 1 attack, the proportion of attacks with a median pain score ≥ 7, and the proportion of patients with ≥ 1 attack with a median pain score ≥ 7 were all lower in the givosiran group compared with the placebo group (Fig. 5).

**Fig. 6:**
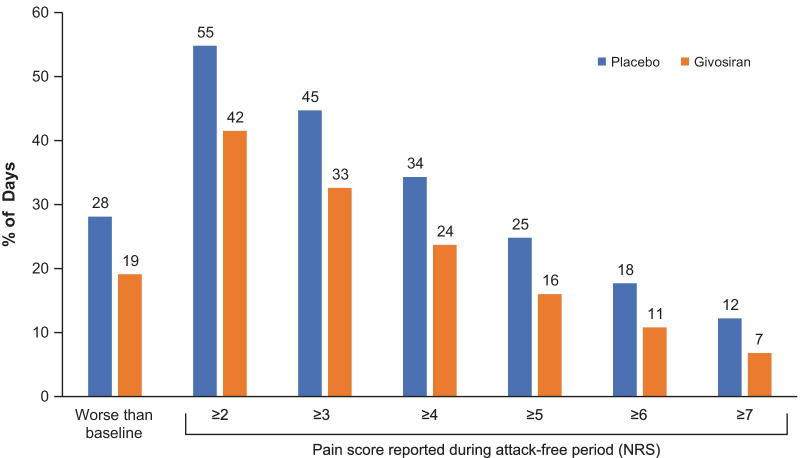
Daily worst pain scores during attack-free periods in ENVISION trial

**Fig. 7:**
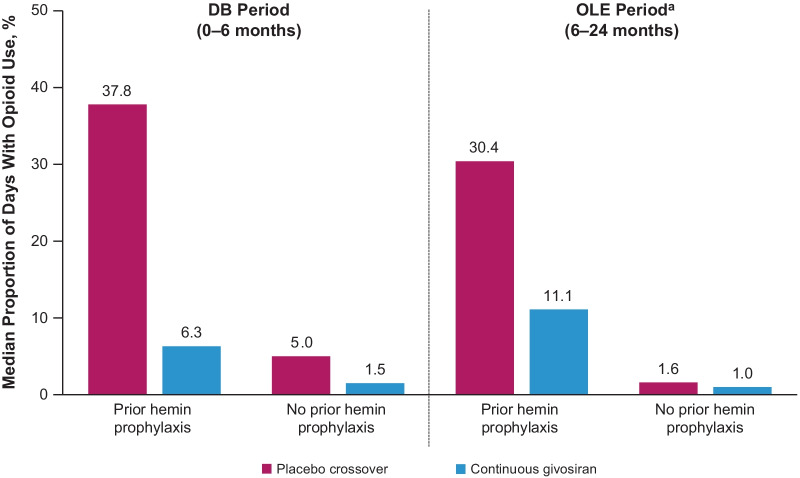
Proportion of days with opioid use in patients with or without prior hemin prophylaxis use. ^a^Analgesic use was collected in an electronic diary up to Month 12

**Fig. 8:**
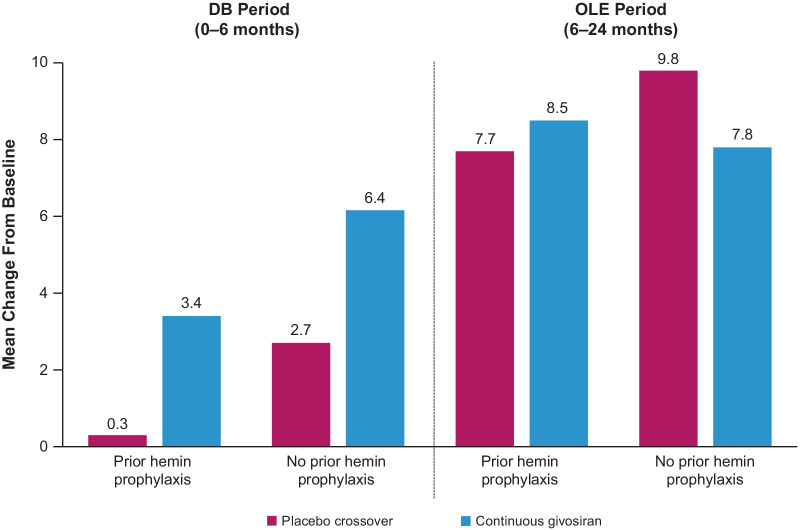
Mean change from baseline in SF-12 PCS scores in patients with or without prior hemin prophylaxis use. Higher scores represent improvement in that summary or domain

AHP patients who received givosiran had reduced pain and analgesic use both during and between attacks compared with those who received placebo. Those in the givosiran group also had fewer days with daily worst pain scores above baseline (Fig. 6). Furthermore, they reported nearly 50% fewer days with severe pain during attack-free periods compared with placebo recipients (proportion of days with a pain score ≥ 7; 7% vs. 12%). Opioid analgesics were used by 73% of patients in the givosiran group and 85% of patients in the placebo group during attacks [38]. Corresponding percentages during attack-free periods were 56% and 70%, respectively [38]. The proportion of days with opioid use was reduced in patients with AHP receiving givosiran compared with placebo, regardless of prior hemin use (Fig. 7). Givosiran treatment was also associated with improved QOL, measured by higher SF-12 PCS scores (Fig. 8).

## Discussion

This study used data from the ENVISION trial to demonstrate the severe disease burden associated with recurrent AHP. At baseline, more than half of ENVISION trial patients were experiencing chronic symptoms between attacks, including nausea, fatigue, and pain, and more than one quarter had been using opioids daily or on most days [35]. We also found a positive correlation between time since diagnosis and AHP disease severity, further highlighting the need to treat patients early with effective therapy. These results showed that the RNAi therapeutic givosiran is effective in patients with recurrent AHP regardless of prior hemin use, and that it reduces analgesic use and pain both during and between porphyria attacks.

Overall disease burden in the ENVISION trial population was similar to that observed in the recent EXPLORE study, a prospective, multinational, natural history study of patients with AHP experiencing recurrent attacks [11]. Eligibility criteria for EXPLORE were similar to the ENVISION trial, with EXPLORE patients needing to have experienced ≥ 3 attacks in the previous 12 months, including ≥ 1 attack requiring hemin or treatment at a hospital or healthcare setting, or be receiving prophylactic treatment to prevent attacks [11]. The median number of attacks experienced in the 12 months before study entry in EXPLORE was 6 [11] compared with 8 in the ENVISION trial [35]. In EXPLORE, 46% of patients reported experiencing chronic porphyria symptoms daily [11] compared with 52% of patients in the ENVISION trial (symptoms daily or on most days) [35]. QOL at baseline, as assessed by EQ VAS, was diminished to a similar extent in EXPLORE (66) and ENVISION (about 63–64) [11]. Of note, rates of psychiatric disorders were higher in ENVISION than in EXPLORE (depression 27% vs. 18%, anxiety 23% vs. 8%, insomnia 18% vs. 12%, respectively) [11].

The proportions of patients with chronic symptoms in both ENVISION and EXPLORE (52–65%) were considerably higher than those reported in earlier US and Swedish observational studies (18–22%) [6, 11, 35, 39]. However, the rate of neuropathy in ENVISION (38%) was similar to that reported in the US observational study (43%) [6], and rates of kidney disease were also similar (25% and 29%) [6], suggesting that disease progression was comparable between studies.

The diagnosis of AHP is frequently delayed for years due to the non-specific nature of AHP symptoms [6]. In the US observational study of patients with symptomatic AHP, the mean time to disease diagnosis was 15 years [6]. Acute attacks in AHP can be difficult to distinguish from other common conditions [5, 40]. Delays in AHP diagnosis are often accompanied by inappropriate treatments for wrongly diagnosed conditions and unnecessary complications for the patient [41, 42]. Recurrent attacks of porphyria may lead to progressive or irreversible neuropathy and prolonged debilitation [2, 11].

Despite the high historical AAR, only about 50% of patients in ENVISION met the European Porphyria Network (EPNET) classification criteria for recurrent attacks, which are used as a marker for disease severity [43]. EPNET defines “recurrent” disease as ≥ 4 attacks in one or more years requiring hospitalization and hemin [43]. EPNET disease severity criteria do not include attacks requiring urgent care or hemin at home and do not include chronic symptoms of AHP. In comparison, the inclusion criteria for ENVISION required patients to have experienced ≥ 2 attacks requiring hospitalization, urgent care, or IV administration of hemin at home in the previous 6 months [35]. By using less-strict criteria for defining recurrent attacks, the ENVISION trial included patients across a range of disease severity in terms of the number of acute attacks and setting of acute attack treatment. This enabled an analysis of the long-term burden of AHP by accounting for multiple measures of disease severity, including chronic symptoms between attacks, pain severity and use of opioid analgesics, QOL and lost days of work, prevalence of comorbidities, prevalence of prophylactic hemin use, and complications associated with hemin prophylaxis [35]. Urgent healthcare visits and use of hemin at home for acute attacks accounted for 63% of historical AAR in the ENVISION trial.

Our study also examined disease burden at baseline according to prior hemin use in ENVISION trial patients and showed that disease burden remained high in patients who had not received hemin prophylaxis; approximately 60% experienced chronic symptoms between attacks, with a median historical AAR of 6 in the placebo group and 8 in the givosiran group.

In ENVISION, the number of porphyria attacks and attack severity were both reduced in patients receiving givosiran compared with those receiving placebo, regardless of prior hemin use. Givosiran recipients had fewer days with daily worst pain scores above baseline than placebo recipients, and nearly 50% fewer days with severe pain during attack-free periods. Pain is one of the key factors associated with diminished QOL among patients with AHP [11]. The SF-12 PCS scores increased by 10.0 and 8.9 points in the open-label extension in the placebo crossover and continuous givosiran groups, respectively. The proportion of patients who used opioid analgesics during attacks was 12% lower in the givosiran group compared with the placebo group during attacks, and 13% lower during attack-free periods. Given that long-term use of opioids is associated with tolerance, dependence, and addiction, a reduction in use of these medications is clinically relevant. Furthermore, evidence for the efficacy of opioids in the management of chronic non–cancer-related pain is limited. A 30-month open-label extension phase of ENVISION was completed in May 2021. Results to date support long-term maintenance of benefit with givosiran.

A strength of our study is that, unlike the observational EXPLORE study [11], all potential porphyria attacks occurring in the ENVISION trial were adjudicated by the investigator. A limitation of our study was the post hoc nature of our analyses and lack of prespecified formal statistical comparisons; therefore, the results should be interpreted carefully.

## Conclusions

In conclusion, the current study highlights the severe disease burden associated with AHP, even in patients with a relatively low rate of attacks, and supports the effectiveness of givosiran for the management of certain acute and chronic porphyria symptoms.

## Data Availability

De-identified individual participant data that support these results will be made available in a secure-access environment 12 months after study completion. Access will be provided contingent upon the approval of a research proposal and the execution of a data sharing agreement.
